# The pathogenic p.Gln319Ter variant is not causing congenital adrenal hyperplasia when inherited in one of the duplicated CYP21A2 genes

**DOI:** 10.3389/fendo.2023.1156616

**Published:** 2023-05-31

**Authors:** Pavlos Fanis, Nicos Skordis, Meropi Toumba, Michalis Picolos, George A. Tanteles, Vassos Neocleous, Leonidas A. Phylactou

**Affiliations:** ^1^Department of Molecular Genetics, Function and Therapy, The Cyprus Institute of Neurology and Genetics, Nicosia, Cyprus; ^2^Division of Paediatric Endocrinology, Paedi Center for specialized Paediatrics, Nicosia, Cyprus; ^3^School of Medicine, University of Nicosia, Nicosia, Cyprus; ^4^Department of Paediatrics, Paediatric Endocrinology Clinic, Aretaeio Hospital, Nicosia, Cyprus; ^5^Department of Endocrinology, Alithias Endocrinology Center, Nicosia, Cyprus; ^6^Department of Clinical Genetics, The Cyprus Institute of Neurology and Genetics, Nicosia, Cyprus

**Keywords:** CYP21A2, 21-hyrdroxylase deficiency, RCCX, gene duplications, CAH

## Abstract

**Objective:**

The study aimed to identify the pathogenic status of p.Gln319Ter (NM_000500.7: c.955C>T) variant when inherited in a single *CYP21A2* gene (bimodular RCCX haplotype) and to discriminate between a non-causing congenital adrenal hyperplasia (CAH) allele when inherited in a duplicated and functional *CYP21A2* gene context (trimodular RCCX haplotype).

**Methods:**

38 females and 8 males with hyperandrogenemia, previously screened by sequencing and identified as carriers for the pathogenic p.Gln319Ter, were herein tested by multiplex ligation-dependent probe amplification (MLPA) and a real-time PCR Copy number Variation (CNV) assay.

**Results:**

Both MLPA and real-time PCR CNV analyses confirmed a bimodular and pathogenic RCCX haplotype with a single *CYP21A2* in 19/46 (41.30%) p.Gln319Ter carriers and who in parallel all shared elevated 17-OHP levels. The remaining 27 individuals that also carried the p.Gln319Ter exhibited low 17-OHP levels as a result of their carriership of a duplicated *CYP21A2* with a trimodular RCCX haplotype. Interestingly, all of these individuals also carried in linkage disequilibrium with p.Gln319Ter two single nucleotide polymorphisms, the c.293-79G>A (*rs114414746*) in intron 2 and the c.*12C>T (*rs150697472*) in the 3’-UTR. Therefore, these variants can be used to distinguish between pathogenic and non-pathogenic genomic contexts of the c.955T (p.Gln319) in the genetic diagnosis of congenital adrenal hyperplasia (CAH).

**Conclusion:**

The employed methodologies identified a considerable number of individuals with non-pathogenic p.Gln319Ter from the individuals that typically carry the pathogenic p.Gln319Ter in a single *CYP21A2*. Therefore, it is extremely important the detection of such haplotypes for the prenatal diagnosis, treatment and genetic counseling in patients with CAH.

## Introduction

Congenital adrenal Hyperplasia (CAH) represents a group of autosomal recessive defects each of which is caused by the impairment of several enzymes that are involved in cortisol biosynthesis (1, 2). The most frequent form of CAH is 21-hydroxylase deficiency (21-OHD) (90–95% of cases) followed by 11β-hydroxylase deficiency (11β-OHD) (∼5% of cases) and other rarer forms (3). The Non Classic (NC)-CAH which is the mild form of the disorder is more frequently observed in females (4). Males with NC-CAH are diagnosed significantly less frequently than females due to less frequently presented and recognized signs of androgen excess (4, 5). The multiallelic and tandem RCCX (**R**=*RP1* also known as *STK19*, **C**=*C4* or complement Component C4, **C**=*CYP21A2* or steroid 21-hydroxylase, and **X**=*TNXB* or tenascin-X) copy number variation (CNV) complex exists in the major histocompatibility complex (MHC) class III region on chromosome 6p21.3. The RCCX complex typically comprises of one (monomodular), two (bimodular) or three (trimodular) segments with an incidence of 15%, 75% and 10% in Europeans, respectively (6, 7). Each segment itself is also quite multipart since it contains a series of four genes; the serine/threonine kinase 19 (*STK19*), the complement 4 (*C4*), the steroid 21-hydroxylase (*CYP21A2*), the tenascin-X (*TNX*) and their corresponding pseudogenes (8). The majority of pathogenic variants in 21-OHD cases are transferred to the *CYP21A2* gene by small conversions from the *CYP21A1P* pseudogene as a result of unequal crossover during meiosis (6, 9–13). The complex genetic structure of the RCCX CNV complicates further the genetic diagnosis of 21-hydroxylase deficiency, in particular when a haplotypic structure with the segment harboring two *CYP21A2* gene copies and one *CYP21A1P* pseudogene copy is involved [6]. A very characteristic example of a haplotypic RCCX CNV structure containing three distinct segments (i.e the trimodular haplotype) with one of them carrying the deleterious p.Gln319Ter variant is quite often observed and confuses the genetic diagnosis of CAH (6, 14–17). Numerous studies in the Mediterranean region identified p.Gln319Ter pathogenic variant as quite frequent (18–20). Similarly, in a previous study by our group three hundred unrelated subjects (150 males and 150 females) from the general population of Cyprus were screened for pathogenic variants in the *CYP21A2* gene and identified p.Gln319Ter variant among six others as the second most frequent (21). In this same study, an estimated *CYP21A2* carrier frequency of 9.83% for the Cypriot population was also reported, with the mild p.Val282Leu being the most frequent (4.3%), followed by p.Gln319Ter (2.5%), p.Pro454Ser (1.33%), p.Val305Met (0.83%), p.Pro483Ser (0.67%) and p.Met284Val (0.17%) (21).

Since the first report by Globerman et al. (22) where a stop codon in exon 8 of the *CYP21A2* gene at position 319 (p.Gln319Ter) was identified, numerous other followed (4, 23–25). Individuals homozygous for this mutation have the severe salt-wasting (SW) form of CAH and no enzymatic activity (22, 26). Several other reports have also demonstrated that heterozygosity for the p.Gln319Ter variant can be an issue especially in females and that exhibit the milder NC-CAH form (27–29).

In the present study, we used two different quantitative methods, a multiplex ligation-dependent probe amplification (MLPA) methodology and a real-time PCR assay to test for the presence of a duplicated *CYP21A2* gene. We have re-examined using the above-mentioned two quantitative methodologies, 46 heterozygous and/or compound heterozygous individuals and that were initially screened by Sanger and were identified as carriers of the severe p.Gln319Ter mutation. Our findings demonstrated that a lower frequency with the duplicated *CYP21A2* gene trimodular RCCX haplotype exists in the Cypriot cohort under investigation. Furthermore, in the same cohort a lower incidence of the pathogenic p.Gln319Ter mutation is carried in a RCCX haplotype with one copy of the *CYP21A2* gene. This finding demonstrates the necessity of using in addition to the traditional Sanger sequencing also other contemporary methodologies that are needed for the more precise and accurate diagnosis of CAH.

## Materials and methods

### Study subjects

In the present study a sample collection of 46 (38 females and 8 males) Cypriot patients with clinical hyperandrogenism. According to age, patients were diagnosed with clinical signs of hyperandrogenemia. Pre-pubertal patients; girls younger than 8 years of age and boys younger than 9 years of age presented with premature adrenarche (pubic or/and underarm hair, body odor, acne spots), and/or bone age advancement, and/or hypertrophic clitoris. Patients in post-pubertal ages presented with severe acne, hirsutism, infrequent menstrual bleeding, voice hoarseness and/or male pattern balding. All patients underwent measurements of basal and/or stimulated 17-OH Progesterone (P) levels. Sanger sequencing was previously performed to all patients with clinical hyperandrogenaemia either with normal or mildly elevated basal or/and stimulated levels of 17-OH Progesterone (P) and irrespectively of the levels of the other androgens (testosterone, androstenedione and DHEA-S). All patients included, were previously identified with Sanger sequencing as carriers for the p.Gln319Ter in the *CYP21A2* gene, were also subjected to further genetic analysis as described below using two other quantitative PCR based methodologies. Parental samples were not available for analysis. Informed consent was obtained from all adult patients and the parents or guardians of the minors. The project was approved by the Cyprus National Ethics Committee (EEBK/EΠ/2016/28) and all methods were performed in accordance with the relevant guidelines and regulations and the Declaration of Helsinki of 1964 and its later amendments.

### Copy number variation analysis by MLPA and real-time PCR assay

All 46 samples were further re-examined using two quantitative PCR based methodologies as per the recommendation of the CAH Best Practice Guidelines (30). The two methodologies used, were the multiplex ligation-dependent probe amplification (MLPA) as previously described (12, 31) using the SALSA MLPA CAH P050-D1 Probemix (MRC-Holland, Amsterdam, the Netherlands) and the commercial CAH Real Fast CNV Assay real-time PCR according to manufacturer’s instructions (ViennaLab Diagnostics GmbH, Austria). The Real-time PCR was performed on QuantStudio 3 (Applied Biosystems, Foster City, CA, USA). Both methods intended to test for the presence of a duplicated *CYP21A2* gene.

### Sanger sequencing analysis

The complete *CYP21A2* gene downstream of the *TNXB* gene was amplified for all 46 samples using the primers CYP779f (5’-AGGTGGGCTGTTTTCCTTTCA-3’) and Tena32F (5’-CTGTGCCTGGCTATAGCAAGC-3’) according to the protocol of Lee et al. (32), generating an 8.5 Kb product that was sequenced with specific internal primers. Specifically, the PCR reaction mixture was performed using the PrimeStar GXL DNA polymerase kit (Takara-Bio, Shiga, Japan) at a final volume of 50 μl and contained 10 μl of Primestar GLX buffer (5x), 4 μl dNTP mix (2.5 mM), 1 μl of each primer (10μM), 2 μl PrimeSTAR GXL DNA Polymerase (1.25 U/μl) and 250 ng of genomic DNA. Amplification was performed with an initial denaturing temperature at 98°C for 5min, followed by 32 cycles of denaturation (98°C, 10sec), annealing (60°C, 30sec), extension (68°C, 3min), with a final extension at 68°C for 7min. For the 27 samples identified by MLPA and Real-time PCR assay to have three copies of the *CYP21A2* gene, we also amplified and sequenced the complete the *CYP21A2* gene downstream of the *TNXA* gene. To achieve this we initially amplify the gene using the CYP779f and XA-36F (5’-GGACCCAGAAACTCCAGGTGG-3’) primers according to the protocol of Tsai et al. (17), generating a 6.1 Kb product. The PCR mixture was the same as above and the amplification was performed with an initial denaturing temperature at 98°C for 5min, followed by 28 cycles of denaturation (98°C, 10sec), extension (68°C, 3min), with a final extension at 68°C for 7min. Next, 20 μl of the PCR product were used for restriction enzyme digestion with TaqI at 65°C for 2 hrs. The whole digestion reaction was loaded on a 1% agarose gel and the 3.7 Kb fragment was extracted and purified using the NucleoSpin gel and PCR cleanup kit (Macherey-Nagel, Düren, Germany) and then sequenced with specific internal primers. The sequencing results were analysed using the Seqscape v3.0 software (Applied Biosystems, Foster City, CA).

## Results

### *CYP21A2* gene p.Gln319Ter pathogenic variant

In the course of Sanger sequencing for the *CYP21A2* gene downstream of the *TNXB* gene, the 46 patients with hyperandrogenemia were previously identified by our group as carriers for the p.Gln319Ter pathogenic variant (12, 31). Thirty-five of the above patients carried p.Gln319Ter in heterozygosity and the remaining eleven in compound heterozygosity with another *CYP21A2* pathogenic causing variant (**Table 1**). As described below the employment of MLPA and the quantitative Real-time PCR CNV assay delineated the pathogenic p.Gln319Ter variants.

**Table 1:** RCCX copy number variation haplotypes in 46 individuals with hyperandrogenemia all found to carry the p.Gln319Ter pathogenic variant in the *CYP21A2* gene.

	RCCX haplotype with a single *CYP21A2* gene & 17-OHP* levels (nmol/l)	Trimodular RCCX haplotype with two (duplicated) *CYP21A2* genes & 17-OHP* levels (nmol/l)
**Individuals with p.Gln319Ter (n=46)**	n= 19 (41.30%) 17-OHP: Average = 43.9; Range = 28.3 – 63.7	n=27 (58.7%) 17-OHP: Average = 4.3; Range = 2.7 – 8.27
**Heterozygotes with p.Gln319Ter (n=35)**	11 (31.4%) 17-OHP: Average = 37.1; Range = 28.3 – 45.4	24 (68.6%) 17-OHP: Average = 4.3; Range = 2.7 – 8.27
**Individuals with p.Gln319Ter in Compound heterozygosity with another *CYP21A2* mutation (n=11)**	8 (72.7%) 17-OHP: Average = 57.9; Range = 43.6 – 88.7	3 (27.3%) 17-OHP: Average = 4.2; Range = 3.2 – 5.6
**Individuals with p.Gln319Ter in linkage disequilibrium with rs114414746 & rs150697472 of the *CYP21A2* gene (n=27)**	0 (0%)	27 (100%)

*Normal 17-OHP levels = 0.6-6.7 nmol/l.

### MLPA analysis for the detection of *CYP21A2* gene duplication

**Figure 1:**
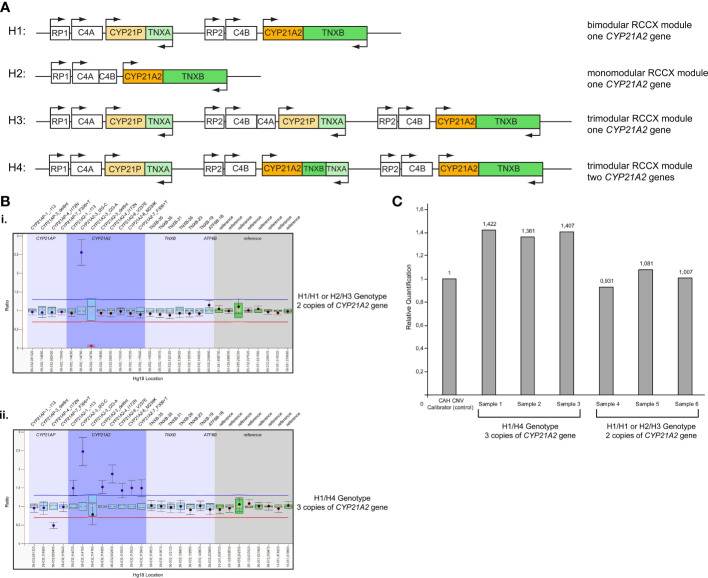
**(A)** Schematic representation of the four different haplotypes identified in the present study. H1: Haplotype 1, bimodular RCCX module with one *CYP21A2* gene; H2: Haplotype 2, monomodular RCCX module with one *CYP21A2* gene; H3: Haplotype 3, trimodular RCCX module with one *CYP21A2* gene; H4: Haplotype 4, trimodular RCCX module with two *CYP21A2* genes. **(B)** MLPA representative ratio charts of patients identified as carriers for the pathogenic variant p.Gln319Ter showing the i) H1/H1 or H2/H3 genotype and the ii) H1/H4 genotype on chromosome 6p21.3. **(C)** Representative CAH Real Fast CNV Assay of patients identified as carriers for the pathogenic variant p.Gln319Ter showing the H1/H4 genotype (samples 1-3) and the H1/H1 or H2/H3 genotype (samples 4-6). CAH CNV calibrator (Vienna Lab) used as control with ratio=1.

Further analysis by MLPA of the 46 patients initially identified by Sanger sequencing as carriers for the p.Gln319Ter, revealed the presence of four haplotypes that were combined in different genotypes. The four haplotypes were H1: bimodular RCCX module with one *CYP21A2* gene, H2: monomodular RCCX module with one *CYP21A2* gene, H3: trimodular RCCX module with one *CYP21A2* gene and H4: trimodular RCCX module with two *CYP21A2* genes (**Figure 1A**). Interestingly, only 19 (41.30%) of the patients were found to carry two haplotypes containing one *CYP21A2* gene each (**Table 1**, **Supplementary Table 1**, **Figure 1A, B**(i)). Eleven of these 19 hyperandrogenic patients carried p.Gln319Ter in the heterozygous state with the remaining eight to carry it in compound heterozygosity with another *CYP21A2* causing variant. The remaining 27 patients that carried the p.Gln319Ter variant in the heterozygous form shared the trimodular H4 haplotype with two *CYP21A2* genes and a single *CYP21A1P* pseudogene [**Table 1**, **Supplementary Table 1**, **Figure 1A, B(ii)**].

### Real-time PCR CNV assay for the detection of *CYP21A2* gene duplication

All 46 samples from the unrelated patients with hyperandrogenemia were further re-analyzed using the commercial real-time PCR CAH Real Fast CNV Assay (Vienna Lab) so as to re-confirm the presence or absence of the duplicated *CYP21A2* genes obtained by MLPA. Likewise, the real-time PCR assay confirmed the presence of a duplicated *CYP21A2* in the same 19 patients that were detected with MLPA (**Figure 1C**).

### Molecular characterization of the trimodular H4 haplotype with two *CYP21A2* genes and a single *CYP21A1P* pseudogene

**Figure 2:**
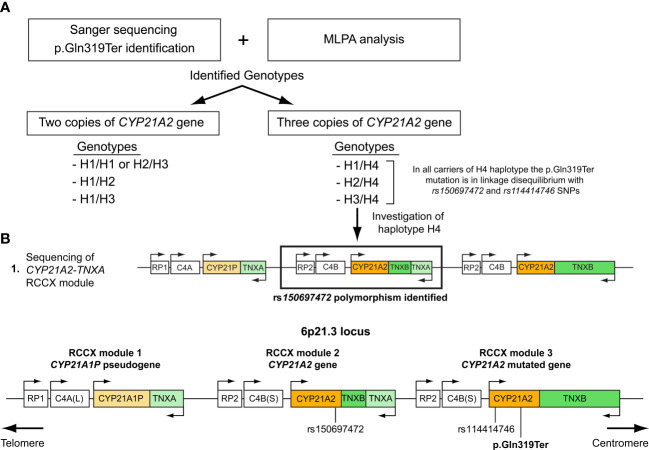
**(A)** Illustration of the procedure followed in this study to genotype the samples carrying the p.Gln319Ter mutation. **(B)** Schematic representation of the trimodular RCCX haplotype on chromosome 6p21.3 consisting of three segments. RCCX module 1 containing the *CYP21A1P* pseudogene and RCCX modules 2 and 3 containing the *CYP21A2* gene. Both the p.Gln319Ter mutation and the *rs114414746* (NM_000500.9: c.293-79G>A) polymorphism in intron 2 of the *CYP21A2* gene are located in the third non-functional segment, while the *rs150697472* (NM_000500.9: c.*12C>T) is located in the 3’UTR of the second functional segment.

All 27 subjects who shared the trimodular H4 haplotype were also found to carry in complete linkage disequilibrium (LD) with the p.Gln319Ter the SNPs *rs114414746* (NM_000500.9: c.293-79G>A) in intron 2 and the *rs150697472* (NM_000500.9: c.*12C>T) in the 3’-UTR. To investigate this further, we specifically sequence the *CYP21A2* gene, which is located downstream of the *TNXA* gene. Sequencing revealed that the *CYP21A2* gene downstream of the *TNXA* gene contains the SNP *rs150697472* but not p.Gln319Ter and SNP *rs114414746* (**Figure 2A**). Combined with the results of sequencing of the *CYP21A2* gene downstream of the *TNXB* gene, the middle segment of the H4 trimodular haplotype harbors the SNP c.*12T (*rs150697472*), while the third segment harbors the two variants c.293-79A (*rs114414746*) and the c.955T (p.Gln319) (**Figure 2B**).

### Biochemical and genotypic correlation of the identified patients with the p.Gln319Ter variant

Nineteen (12 females and 7 males) patients who were found to have two copies of the *CYP21A2* gene and carry the pathogenic p.Gln319Ter variant either in the heterozygous or compound heterozygous state and had elevated 17-OHP levels (Average = 43.9, Range = 28.3 – 63.7). It is noted that normal levels of 17-OHP are 0.6 - 6.7 nmol/l. The remaining 27 subjects that carried in the trimodular H4 haplotype the p.Gln319Ter variant shared lower 17-OHP levels (Average = 4.3, Range = 2.7 – 8.27) (**Table 1**). As predicted, the heterozygote patients with the bimodular RCCX haplotype carrying the pathogenic p.Gln319Ter exhibited lower 17-OHP values (Average = 37.1, Range = 28.3 – 45.4) when compared to the values of patients (n=8) that shared p.Gln319Ter in compound heterozygosity with another *CYP21A2* pathogenic variant (Average = 57.9, Range = 43.6 – 88.7) (**Table 1**). It should be noted that six out of eight of the above patients that carried p.Gln319Ter in a bimodular RCCX haplotype also carried in compound heterozygosity the p.Val282Leu mutation. All six (five females and one male) of them were classified as non-classic (NC) CAH and their 17-OHP levels ranged between 43.6-61.9 nmol/l. The remaining two female patients of the above cohort were respectively found to carry in their second allele the severe IVS-13A/C>G and the milder p.Pro454Ser mutations. As a result of the severe IVS-13A/C>G/p.Gln319Ter genotype the neonate female exhibited higher 17-OHP levels (88.7 nmol/l) and was born with ambiguous genitalia, therefore was classified with the severe Salt-Wasting (SW) CAH form. Lastly, the girl identified with the p.Gln319Ter/p.Pro454Ser genotype exhibited lower 17-OHP levels (52.4 nmol/l) and phenotypically was classified with the NC-CAH form.

## Discussion

Duplications of the *CYP21A2* gene have been reported as a result of the less frequent RCCX CNV trimodular haplotype that harbors one copy of the *CYP21A1P* pseudogene and two copies of the *CYP21A2* gene, with the latter to also harbor the p.Gln319Ter variant in 2-7% of the general population (15, 16, 33, 34). In the present study genotyping of 46 individuals with p.Gln319Ter variant using the commercially available MLPA and the Real-time PCR CNV Assay, demonstrated that the majority of these individuals (27/46; 58.7%) carried the p.Gln319Ter aberration on the allele that harbors the duplicated *CYP21A2* gene. It should be noted, that all 46 individuals of the present study were referred to our laboratory for genetic investigation since they were identified with clinical signs of hyperandrogenemia. As evidenced by the extended haplotype analysis from the MLPA and the real time PCR analyses of the present study, the p.Gln319Ter aberration was found in a genomic non-pathogenic context in 58.7% of the tested individuals as a result of the presence of the trimodular RCCX haplotype. Consequently, this haplotype format is not accountable for causing their clinical manifestations of hyperandrogenemia. Despite the fact that several recent studies likewise identified p.Gln319Ter often to be associated with an RCCX trimodular and duplicated *CYP21A2* haplotype (33, 35), the unavailability of parental samples for more explicit haplotype analyses would be more appropriate to hypothesize this as described previously (36) (37). A future study aiming to determine the actual frequency of the trimodular non-pathogenic haplotype harboring the p.Gln319Ter variant in a cohort of clinically unaffected Cypriot individuals will undoubtedly contribute towards the avoidance of false-positive genotyping and assist for the more precise CAH diagnosis in the island.

It’s worth mentioning that for the more accurate CAH diagnosis the quantitative based methodologies such as the MLPA and the real-time PCR assay are absolutely essential and in the present study were proven so. The usage of both of these methodologies enabled us to clarify the carrier status of the p.Gln319Ter in the trimodular non-CAH causing allele from the bimodular CAH allele. Moreover, we have also noticed that the majority (8/11; 72.7%) of the compound heterozygote patients for the p.Gln319Ter indeed shared the bimodular and pathogenic RCCX haplotype with a single *CYP21A2* gene (**Table 1**). On the contrary, the majority (24/35; 68.6%) of the non-pathogenic heterozygotes for the p.Gln319Ter variant shared the trimodular RCCX non-pathogenic haplotype as a result of the presence of two *CYP21A2* gene copies (**Table 1**, **Figure 2B**).

Lastly, our observation that all carriers of the duplicated *CYP21A2* gene with the p.Gln319Ter variant were in linkage disequilibrium with two infrequent SNPs, the c.293-79A (*rs114414746*) in intron 2 and the c.*12T (*rs150697472*) in the 3’-UTR (**Table 1**), and the fact that was in agreement with an earlier report by Kleinle et al. (33) reveals the importance of analyzing the *CYP21A2* untranslated regions (**Figure 2B**). In a recent report by Doleschall et al., the sequences of the duplicated *CYP21A2* genes (trimodular RCCX haplotype) were defined (8). More precisely, the *CYP21A2* gene in the middle segment of the trimodular haplotype harbored the c.*12T (*rs150697472*) SNP, whereas the *CYP21A2* gene in the 3’ segment harbored both the c.293-79A (*rs114414746*) SNP and the c.955T (p.Gln319) mutation. This finding in combination with earlier studies in healthy subjects of European descent (Spanish and Italian) (15, 35), and the evidenced by the present study (**Figure 2**) can be used to distinguish between pathogenic and non-pathogenic genomic contexts of the c.955C>T (p.Gln319Ter) in the genetic diagnosis of CAH.

## Conclusions

In conclusion, the absence of solid correlation between genotype and phenotype observed in many individuals with clinical signs of hyperandrogenemia and CAH can largely be explained with the identification of the rare RCCX CNV haplotypes, therefore the need of their assessment is vital for the precise molecular diagnosis of 21-hydroxylase deficiency.

## Data availability statement

The original contributions presented in the study are included in the article/**Supplementary Material**. Further inquiries can be directed to the corresponding author.

## Ethics statement

The studies involving human participants were reviewed and approved by Cyprus National Ethics Committee (EEBK/EΠ/2016/28). Written informed consent to participate in this study was provided by the participants’ legal guardian/next of kin.

## Author contributions

PF conceptualized and designed the study, interpreted data and performed the laboratory experiments. NS, MT, MP and GT performed clinical investigation and enrolled patients. VN conceptualized the study, interpreted data, drafted and revised the manuscript. LP conceptualized the study and revised the final version of the manuscript. All authors contributed to the article and approved the submitted version.
