# Supplementary material for: The pathogenic p.Gln319Ter variant is not causing congenital adrenal hyperplasia when inherited in one of the duplicated CYP21A2 genes
Source: Front Endocrinol (Lausanne). 2023 May 31;14:1156616. doi: 10.3389/fendo.2023.1156616 (PMC10266209; doi:10.3389/fendo.2023.1156616)
Supplement: Supplementary file 1 [file Table_1.docx]

**Supplementary Table 1**. Sanger Sequencing, MLPA results and genotypic analysis of the samples carrying the p.Gln319Ter mutation. For each case, the copy number of the *CYP21A2* gene and *CYP21A1P* pseudogene is indicated. Samples shared the H4 trimodular haplotype are highlighted with yellow color.

| **Patients** | **MUTATIONS IDENTIFIED by SANGER SEQUENCING** | **MLPA RESULT** | | **Genotype** |
| --- | --- | --- | --- | --- |
|  |  | **Number of *CYP21A1P* copies** | **Number of *CYP21A2* copies** | **(Haplotype A/Haplotype B)** |
|  |  |  |  |  |
| 1 | p.Gln319Ter Heterozygote | 2 | 2 | H1/H1 or H2/H3 |
| 2 | p.Val282Leu/p.Gln319Ter | 3 | 2 | H1/H3 |
| 3 | p.Gln319Ter Heterozygote | 2 | 3 | H1/H4 |
| 4 | p.Gln319Ter Heterozygote | 2 | 3 | H1/H4 |
| 5 | p.Gln319Ter Heterozygote | 2 | 3 | H1/H4 |
| 6 | p.Gln319Ter Heterozygote | 2 | 3 | H1/H4 |
| 7 | p.Val282Leu/p.Gln319Ter | 3 | 2 | H1/H3 |
| 8 | p.Gln319Ter Heterozygote | 2 | 2 | H1/H1 or H2/H3 |
| 9 | p.Gln319Ter Heterozygote | 2 | 3 | H1/H4 |
| 10 | p.Gln319Ter Heterozygote | 2 | 3 | H1/H4 |
| 11 | p.Gln319Ter/p.Pro454Ser | 2 | 3 | H1/H4 |
| 12 | p.Gln319Ter Heterozygote | 2 | 2 | H1/H1 or H2/H3 |
| 13 | p.Gln319Ter Heterozygote | 2 | 2 | H1/H1 or H2/H3 |
| 14 | p.Gln319Ter Heterozygote | 2 | 2 | H1/H1 or H2/H3 |
| 15 | p.Gln319Ter/p.Val305Met | 2 | 3 | H1/H4 |
| 16 | p.Gln319Ter /p.Pro483Ser | 2 | 3 | H1/H4 |
| 17 | p.Gln319Ter Heterozygote | 2 | 3 | H1/H4 |
| 18 | p.Gln319Ter Heterozygote | 2 | 3 | H1/H4 |
| 19 | p.Gln319Ter Heterozygote | 2 | 3 | H1/H4 |
| 20 | p.Val282Leu/p.Gln319Ter | 3 | 2 | H1/H3 |
| 21 | p.Val282Leu/p.Gln319Ter | 3 | 2 | H1/H3 |
| 22 | p.Gln319Ter Heterozygote | 2 | 2 | H1/H1 or H2/H3 |
| 23 | p.Gln319Ter Heterozygote | 2 | 3 | H1/H4 |
| 24 | p.Gln319Ter Heterozygote | 2 | 3 | H1/H4 |
| 25 | p.Val282Leu/p.Gln319Ter | 3 | 2 | H1/H3 |
| 26 | IVS2-13A/C>G/p.Gln319Ter | 2 | 2 | H1/H1 or H2/H3 |
| 27 | p.Gln319Ter Heterozygote | 2 | 3 | H1/H4 |
| 28 | p.Gln319Ter Heterozygote | 2 | 3 | H1/H4 |
| 29 | p.Gln319Ter Heterozygote | 2 | 2 | H1/H1 or H2/H3 |
| 30 | p.Gln319Ter Heterozygote | 2 | 2 | H1/H1 or H2/H3 |
| 31 | p.Gln319Ter Heterozygote | 2 | 3 | H1/H4 |
| 32 | p.Gln319Ter Heterozygote | 1 | 2 | H1/H2 |
| 33 | p.Gln319Ter Heterozygote | 3 | 2 | H1/H3 |
| 34 | p.Gln319Ter Heterozygote | 2 | 3 | H1/H4 |
| 35 | p.Gln319Ter Heterozygote | 2 | 2 | H1/H1 or H2/H3 |
| 36 | p.Val282Leu/p.Gln319Ter | 3 | 2 | H1/H3 |
| 37 | p.Gln319Ter Heterozygote | 2 | 3 | H1/H4 |
| 38 | p.Gln319Ter/p.Pro454Ser | 2 | 2 | H1/H1 or H2/H3 |
| 39 | p.Gln319Ter Heterozygote | 2 | 3 | H1/H4 |
| 40 | p.Gln319Ter Heterozygote | 1 | 3 | H2/H4 |
| 41 | p.Gln319Ter Heterozygote | 2 | 3 | H1/H4 |
| 42 | p.Gln319Ter Heterozygote | 2 | 3 | H1/H4 |
| 43 | p.Gln319Ter Heterozygote | 2 | 3 | H1/H4 |
| 44 | p.Gln319Ter Heterozygote | 3 | 3 | H3/H4 |
| 45 | p.Gln319Ter Heterozygote | 2 | 3 | H1/H4 |
| 46 | p.Gln319Ter Heterozygote | 2 | 3 | H1/H4 |
